# Vortioxetine induces apoptosis and autophagy of gastric cancer AGS cells via the PI3K/AKT pathway

**DOI:** 10.1002/2211-5463.12944

**Published:** 2020-09-26

**Authors:** Gao‐Bo Lv, Ting‐Ting Wang, Hai‐Lin Zhu, Hong‐Ke Wang, Wen Sun, Li‐Feng Zhao

**Affiliations:** ^1^ Department of Anal‐colorectal Surgery Baoji Municipal Central Hospital Baoji China; ^2^ Department of Administration Center Outpatient Baoji Municipal Central Hospital Baoji China; ^3^ Department of Hepatobiliary Pancreatic Surgery Baoji Municipal Central Hospital Baoji China; ^4^ Department of Gastroenterology Baoji Municipal Central Hospital Baoji China; ^5^ Beijing Splinger Institute of Medicine Jinan China

**Keywords:** apoptosis, autophagy, gastric cancer, PI3K/AKT pathway, tumorigenicity, vortioxetine

## Abstract

Vortioxetine is a potent antagonist of the 5‐hydroxytryptamine receptor and serotonin transporter and has been reported to function as an antidepressant in the treatment of major depressive disorder. However, its antitumor effects remain unclear. Here, we examined whether vortioxetine affects the characteristics of GC cells. Cell viability was measured by a colony formation assay and, in addition, cell invasion, migration and apoptosis assays were performed with a transwell assay and a flow cytometry assay. Protein levels were measured by western blotting. We found that vortioxetine inhibited the proliferation, invasion and migration abilities of AGS cells. Additionally, vortioxetine could induce apoptosis and autophagy by increasing the levels of Bax, active caspase‐3/‐9, Beclin‐1 and light chain 3, as well as by downregulating Bcl‐2 and P62. Further investigations indicated that vortioxetine regulated apoptosis and autophagy via activation of the phosphoinositide 3‐kinase/AKT pathway. Taken together, our data suggest that vortioxetine has cytotoxic effects against GC AGS cells as a result of inhibiting proliferation, invasion and migration, as well as by inducing apoptosis and autophagy through the phosphoinositide 3‐kinase/AKT pathway.

## Graphical abstract

Vortioxetine, a potent antagonist of the 5‐hydroxytryptamine receptor and serotonin transporter, is known as a novel antidepressant. However, its functional significance of vortioxetine in tumors, including GC, has not been reported to date. Our finding suggest that vortioxetine has cytotoxic effects against GC AGS cells as a result of inhibiting proliferation, invasion and migration, as well as by inducing apoptosis and autophagy through the phosphoinositide 3‐kinase/AKT pathway.

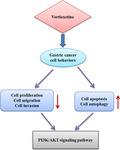

**Abbreviations**
5‐HT: 5‐hydroxytryptamine
ANOVA: analysis of variance
CCK‐8: cell counting kit‐8
GC: gastric cancer
IC_50_: 50% inhibition
LC3: light chain 3
mTOR: mammalian target of rapamycin
PCD: programmed cell death
PI3K: phosphoinositide 3‐kinase

## Introduction

With 1 033 701 new cases and 782 685 deaths worldwide annually, gastric cancer (GC) has become the fifth commonly diagnosed cancer, as well as the second leading cause of malignant cancer death, developing a severe worldwide burden [1, 2]. Currently, despite advances in diagnostic and treatment options, the rates of recurrence and distant metastasis are high and the prognosis for GC patients is poor with respect to the 5‐year survival rate [3, 4, 5, 6]. To date, chemotherapy has been suggested as the first‐line treatment for patients with GC, although GC cells usually appear to be resistant to chemical drugs, with side effects of cytotoxicity [7, 8]. Therefore, the identification of valuable novel drugs is urgently required to improve the curative effect of chemotherapy and the clinical outcome of GC.

As a common form of programmed cell death (PCD), apoptosis has played an important role in regulating the progression of organisms and different cancers [9, 10]. Thus, the molecular mechanism of apoptosis induction has generally been explored for anticancer drugs [11]. Autophagy is known to be a lysosomal‐dependent degradation process that furnishes energy for maintaining metabolism and survival via degradation of long‐lived protein and damaged organelles in cells. It comprises not only a conservative self‐defense pathway for cells, but also a mechanism to distinguish PCD from apoptosis [12, 13, 14]. Usually, autophagy assumes basal levels in cells, although it will be strongly induced under various conditions, such as inflammatory stimulation and hypoxia [15]. Accumulating evidence has demonstrated that autophagy plays an important role in cancer by suppressing the initiation of cancer or promoting cancer growth [16]. Moreover, autophagy is reported to mediate nonapoptotic pathways via a tumor‐suppressive or oncogenic role. For example, the disruption of autophagy may promote tumorigenesis [17] and it could also be induced by anticancer cancer treatment [18, 19]. Given this, investigating the regulatory role of apoptosis and autophagy appears to be particularly important for identifying valuable anticancer agents.

Vortioxetine, known as a novel antidepressant, is approved in the USA and the European Union for the treatment of major depressive disorder [20, 21, 22]. Furthermore, vortioxetine, as a well‐documented antagonist, exerts a potent inhibitory effect on 5‐hydroxytryptamine (5‐HT)_3A_, 5‐HT_7_ receptors and serotonin transporter, whereas it displays partial agonistic properties with respect to 5‐HT_1A_ and 5‐HT_1B_ [23, 24]. Indeed, the 5‐HT receptor is involved in the carcinogenesis of many human tumor types, including bladder, lung, prostate, cholangiocarcinoma and colorectal cancers [25]. 5‐HT receptor overexpression has been found to be linked to the pathogenesis of lung complication of signet ring cell carcinoma of the stomach [26]. Fluoxetine, a specific serotonin reuptake inhibitor, has been reported to significantly induce apoptotic GC cell death [27]. However, the functional significance of vortioxetine in tumors, including GC, has not been reported to date.

Therefore, the present study aimed to evaluate the functional implications of vortioxetine in the pathogenesis of GC cells by investigating its effect on the proliferation, invasion, migration, apoptosis and autophagy capabilities of GC cells.

## Materials and methods

### Cell culture and BafA1 treatment

Human gastric cancer cell line AGS was purchased from the Shanghai Academy of Sciences Cell Bank (Shanghai, China). The cells were cultured in RPMI‐1640 containing 10% fetal bovine serum, 100 U·mL^−1^ penicillin and 0.1 mg·mL^−1^ streptomycin at 37°C in a humidified atmosphere containing 5% CO_2_. After 48 h of culture, AGS cells were treated with vortioxetine and BafA1 (100 nm) in accordance with previous studies [28, 29]).

### Cell counting kit‐8 (CCK‐8) assay

AGS cells were seeded in 96‐well plates and treated with vortioxetine at a series of concentration gradients (0.1, 1, 10, 20 and 50 µm). After 48 h of incubation, the attenuance of each well at 450 nm (*D*_450_) was measured using a microplate reader. Then, cells were incubated with vortioxetine (25.1 µm as the IC_50_) and a normal control (NC, 0.1% DMSO in culture media) for 24, 48 and 72 h.

### Colony formation assay

Cells were placed on a 60‐mm culture dish (500 cells per well) with or without vortioxetine treatment and cultured at 37 °C for 1–2 weeks. Following the appearance of the visible colonies, the cultures were washed with NaCl/Pi and then fixed with 4% paraformaldehyde for 30 min. After removing the fixing solution, the colonies were stained with 0.1% crystal violet for 30 min and counted visually.

### Invasion and migration assays

The invasion and migration assays were analyzed using the transwell assay. For the invasion assay, the upper chamber of 24‐well polycarbonate membrane transwell chambers was initially coated with matrigel (1:6; diluted with serum‐free Dulbecco’s modified Eagle’s medium). Following treatment with vortioxetine for 24 h, 1 × 10^5^ cells in 100 µL suspension were added to the upper chamber, whereas the lower chamber was filed with 500 µL of complete media. After overnight incubation, the cells that failed to pass through the membrane were removed by cotton swabs and the cells attached to the lower surface were fixed 4% paraformaldehyde and stained with 0.1% crystal violet solution for 20 min. The cells that infiltrated the filter were counted in five random fields under microscopy. For the migration assay, cells were seeded at a density of 5 × 10^3^ and the transwell does not need to be coated with glue.

### Flow cytometry analysis

For apoptosis analysis, cells were cultivated in six‐well plates and treated with vortioxetine, harvested using trypsin solution, and stained with annexin V‐fluorescein isothiocyanate and propidium iodide in accordance with the manufacturer’s instructions. Flow cytometric analysis was performed using flowjo (https://www.flowjo.com).

### Western blotting

Cells were seeded in six‐well plates and then lysed with radioimmunoprecipitation assay buffer in an ice bath. Total protein concentration was measured using the bicinchoninic acid assay method. The lysates were loaded into SDS‐PAGE for electrophoresis and then transferred onto a poly(vinylidene difluoride) membrane. Membranes were blocked with 5% skim milk for 1 h, and incubated with primary antibodies against light chain 3 (LC3), AKT, p‐AKT, mTOR, p‐mTOR, cyclin D1, Bcl‐2, Bax, active caspase‐3/‐9 (all dilution 1:1000) and GAPDH (dilution 1:5000) at 4 °C overnight. After washing with Tris‐buffered saline with Tween 20 three times (5 min each time), a secondary goat anti‐rabbit antibody (dilution 1:5000) was added to the membrane and incubated for 1 h. Protein bands were visualized by enhanced chemiluminescence.

### Statistical analysis

All data are reported as the mean ± SD. The statistical analyses were performed using spss, version 18.0 (SPSS Inc., Chicago, IL, USA) and prism, version 5 (GraphPad Software Inc., San Diego, CA, USA). Statistically significant values were compared with Student’s *t*‐tests and analysis of variance (ANOVA) with Dunnett’s and Bonferroni’s post‐hoc tests. *P* < 0.05 was considered statistically significant.

## Results

### Vortioxetine abrogates the proliferative capabilities of GC cells

**Fig. 1:**
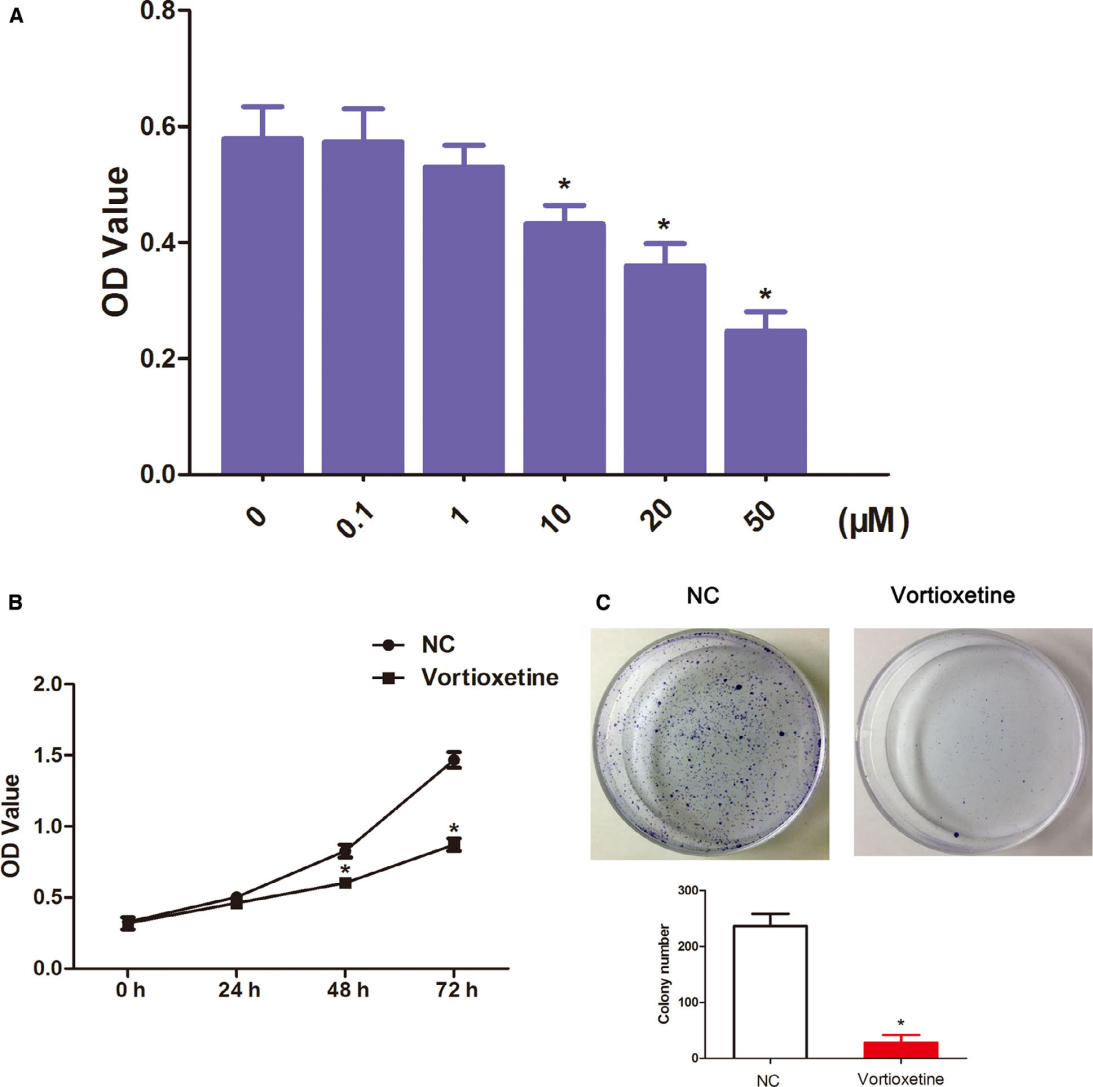
Vortioxetine inhibits the proliferative capacities of AGS cells. (A) The change of AGS cell viability in a dose‐dependent manner was assessed using a CCK‐8 assay. ANOVA with Dunnett’s post‐hoc test was used to investigate differences between multiple groups. (B) The change of AGS cell viability in a time‐dependent manner was assessed using a CCK‐8 assay. Student’s *t*‐test was used to investigate differences between groups. (C) The clonogenic capacity of AGS cell was detected by a colony‐forming assay. Student’s *t*‐test was used to test differences between groups. Data are reported as the mean ± SD (*n* = 3). *P < 0.05.

First, a dose‐dependent experiment was performed. As shown in Fig. 1A, vortioxetine reduced the viability of AGS cells in a concentration‐dependent manner (the concentration of vortioxetine at 0.1, 1, 10, 20 and 50 µm), with 50% inhibition (IC_50_) of 25.1 µm, which was used for the subsequent experiments. We next explored the growth inhibitory effect of vortioxetine on AGS cells by a colony formation assay. As shown in Fig. 1B, the number of clones in the vortioxetine treatment AGS cell group was significantly lower compared to that in the control group (*P* < 0.05), indicating that vortioxetine could inhibit AGS cell growth.

### Vortioxetine impairs the invasiveness and migration abilities of GC cells

**Fig. 2:**
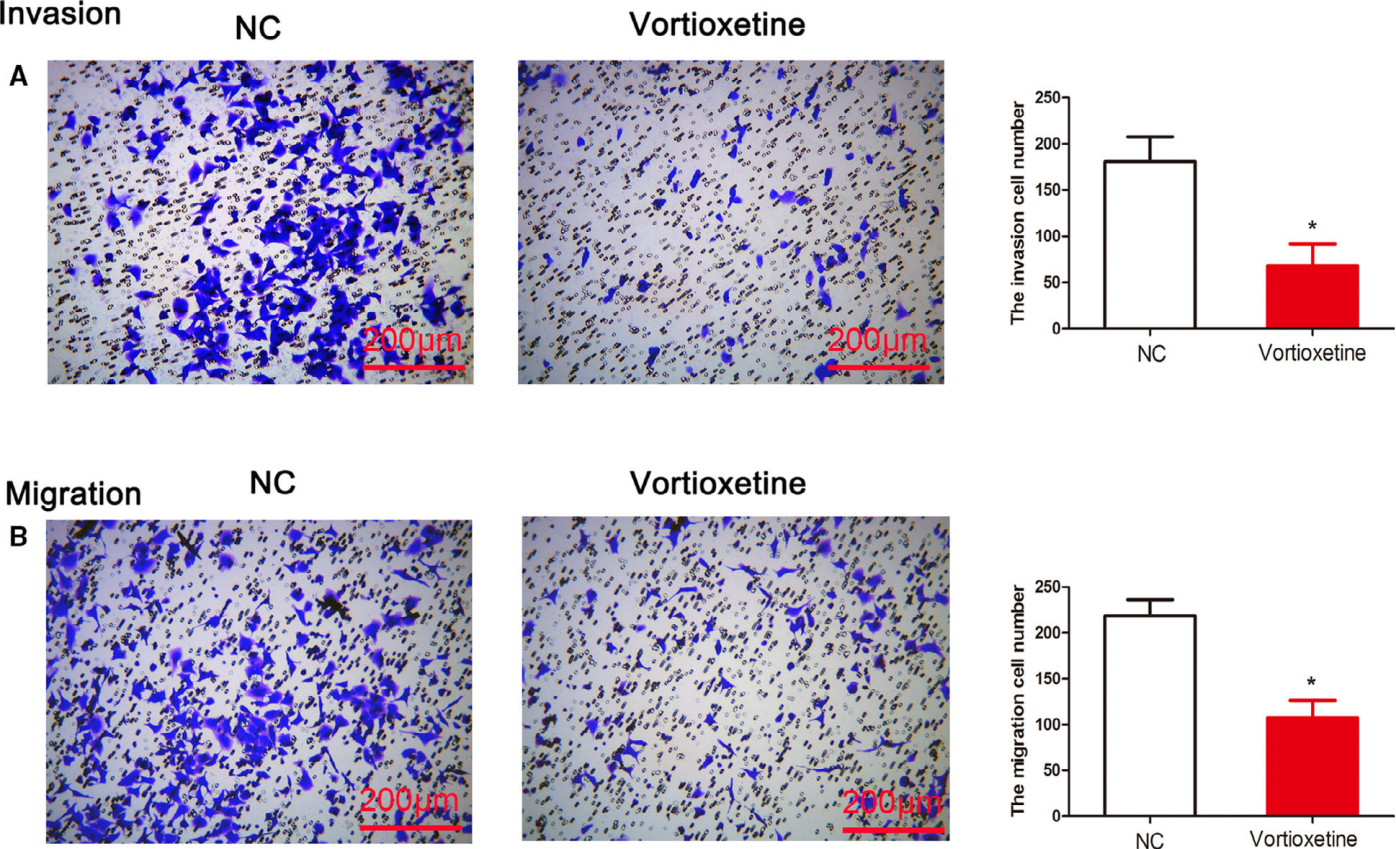
Vortioxetine impairs cell invasion and migration capabilities. (A) Cell invasiveness was assessed by a transwell invasion assay and the number of invading AGS cells was measured. Student’s *t*‐test was used to test differences between groups. (B) Cell migration capabilities were assessed by a transwell migration assay and the number of migrating AGS cells was measured. Student’s *t*‐test was used to test differences between two groups. *P < 0.05 compared to the untreated control. Data are reported as the mean ± SD (*n* = 3). Scale bar = 200 μm

Furthermore, to determine the effect of vortioxetine on the migration and invasiveness of GC cells, a transwell assay was performed. The results of the invasion assay revealed that vortioxetine markedly inhibited the cell invasive abilities of vortioxetine‐treatment compared to the controls (*P* < 0.05) (Fig. 2A). Similarly, migration assays showed that the number of migrated vortioxetine‐treatment cells was significantly lower compared to in controls (*P* < 0.05) (Fig. 2B). Taken together, these results suggested that vortioxetine was cytotoxic to AGS cells and inhibited the tumor progression of GC *in vitro*.

### Vortioxetine induced apoptosis and autophagy of GC cells

**Fig. 3:**
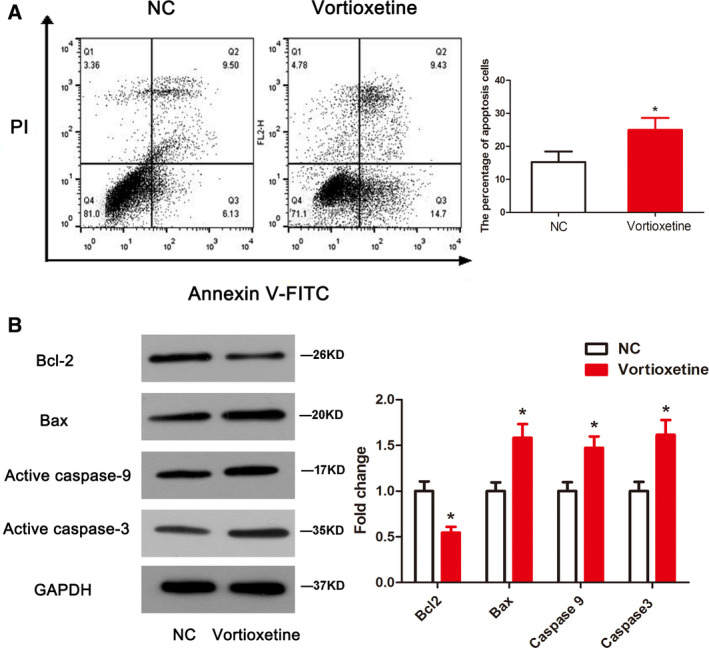
Vortioxetine induces apoptosis in AGS cells. Apoptosis was evaluated by a flow cytometry assay (A) and the protein levels of Bcl‐2, Bax and active caspase‐3/‐9 were measured by western blotting (B). Student’s *t*‐test was used to test differences between groups. *P < 0.05 compared to the untreated control. Data are reported as the mean ± SD (*n* = 3)

To further examine the role of vortioxetine with respect to the suppression of GC cell growth, cell apoptosis was assessed by staining with annexin V‐fluorescein isothiocyanate/propidium iodide. As shown in Fig. 3A, the percentage of apoptotic cells in the vortioxetine‐treatment group was notably higher than that in the control group (*P* < 0.05). Consistent with this finding, the apoptosis‐related proteins levels detected by western blotting suggested that vortioxetine increased the levels of Bax, active caspase‐3 and caspase‐9, whereas it decreased the level of Bcl‐2 (all, *P* < 0.05) (Fig. 3B), indicating that vortioxetine could induce apoptosis in AGS cells that have contributed to cell death.

**Fig. 4:**
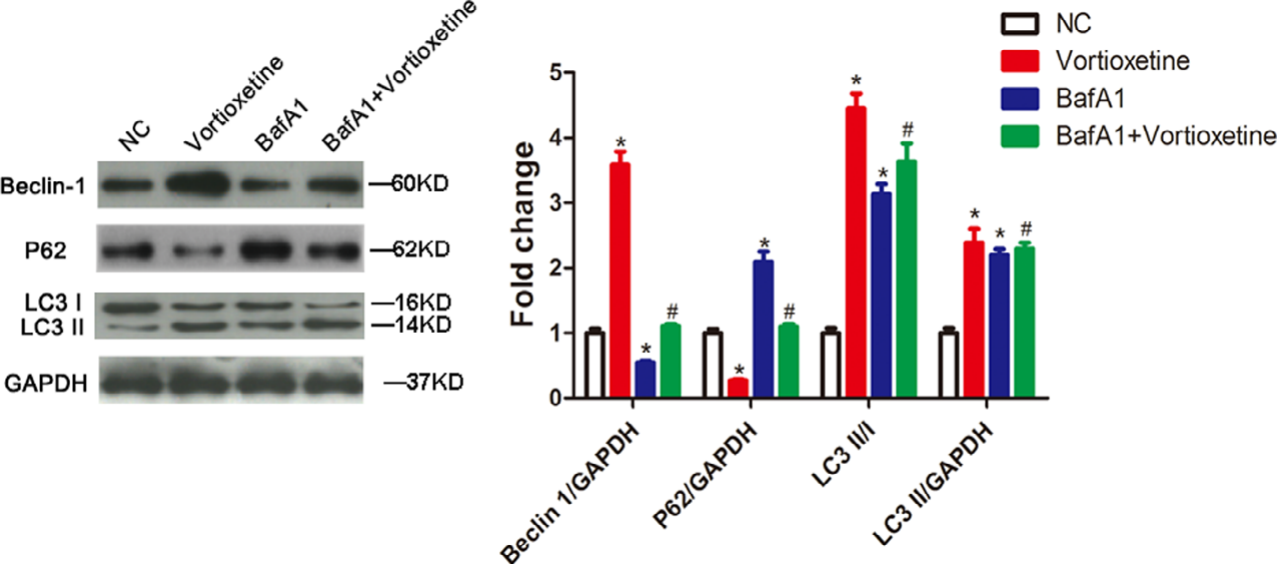
Vortioxetine induces autophagy in AGS cells. Autophagy‐related proteins of Beclin‐1, P62 and LC3 were measured in AGS cells by western blotting. ANOVA with Bonferroni’s post‐hoc test was used to test differences between multiple groups. *P < 0.05 compared to the NC group. #*P* < 0.05 compared to vortioxetine or BafA1. Data are reported as the mean ± SD (*n* = 3).

Furthermore, to investigate whether autophagy was activated in response to vortioxetine exposure, we determined the levels of autophagy‐related proteins including Beclin‐1, P62 and LC3, with/without an autophagy blocker BafA1. The results revealed that the Beclin‐1 expression level, as well as the ratio of LC3 II/I and the level of LC3 II, was significantly higher in AGS cells treated with vortioxetine compared to the controls (both, *P* < 0.05) (Fig. 4). By contrast, compared with control cells, P62 expression was obviously decreased in AGS cells treated with vortioxetine (*P* < 0.05) (Fig. 4). In addition, BafA1 treatment could lead to an accumulation of LC3 II and an increase in the LC3 II/I ratio, with an elevation of the P62 level, compared to the NC group (*P* < 0.05) (Fig. 4). Significantly, co‐treatment of vortioxetine and BafA1 reinforced the LC3 II level and restored the levels of P62 and Beclin‐1 compared to a single treatment group (*P* < 0.05) (Fig. 4). These data indicate that vortioxetine could facilitate AGS cell autophagy.

### Vortioxetine suppressed PI3K/AKT signaling

**Fig. 5:**
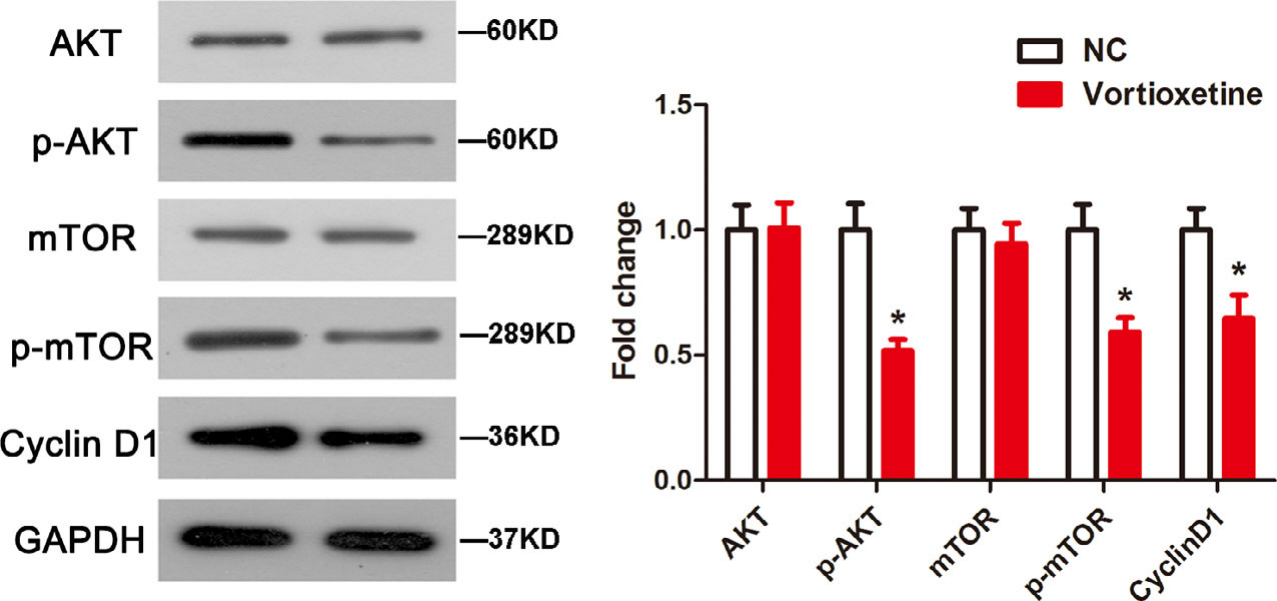
Vortioxetine inhibits the PI3K/Akt pathway in AGS cells. Protein expression was determined by a western blot assay. Student’s *t*‐test was used to test differences between groups. *P < 0.05 compared to the untreated control. Data are reported as the mean ± SD (*n* = 3).

The PI3K/AKT pathway has been identified as the key regulator of apoptosis and autophagy. Therefore, we speculated that vortioxetine might induce apoptosis and autophagy in AGS cells by suppressing the PI3K/AKT signaling pathway. Our results revealed that vortioxetine effectively decreased the phosphorylation levels of AKT and mTOR, as well as cyclin D1 (Fig. 5). The results thus indicate that ectopic vortioxetine might induce apoptosis and autophagy via the activation of the PI3K/AKT pathway in GC cells.

## Discussion

Despite the advances made in the treatment of GC, its prognosis still remains frustratingly poor. Hence, novel and effective therapeutic drugs must be identified to enhance the clinical therapy of GC. The results obtained in the present study indicated that vortioxetine could inhibit GC cell proliferation, migration and invasion. Furthermore, vortioxetine suppressed the growth of GC cells by the induction of apoptosis and autophagy through the PI3K/AKT pathway.

To date, vortioxetine has been the novel antidepressant for treatment of major depressive disorder, although its anti‐tumor effects on GC have not yet been fully investigated. The main biological characteristics of a developing tumor malignancy, including GC, are the abnormal proliferation, migration and invasion of cells, [30, 31]. The results of the cell function assay demonstrated that vortioxetine inhibited tumor progression of GC by inhibiting cell proliferation, invasion and migration.

In the present study, we first confirmed that vortioxetine could induce the apoptosis of AGS cells by regulating apoptosis‐related proteins. Bcl2 protein has been reported to regulate the programmed death of cells as an anti‐apoptotic gene [32]. Furthermore, Bax could also downregulate apoptosis and the mitochondrial membrane in mitochondria as a pro‐apoptotic gene [33]. Caspases, such as cleaved caspase‐3 and caspase‐9, comprise a family of cysteine proteases that contribute to apoptosis and ultimately cell death [34, 35], and Bax and Bcl2 are effective factors for the downstream activation of caspase protein [36]. In the present study, we found that Bax and active caspase‐3/‐9 were highly expressed, whereas Bcl‐2 was decreased in AGS cells treated with vortioxetine.

Accumulating convincing evidence over several decades has shown that the effectiveness of anti‐cancer therapies is not only caused by apoptosis, but also involves autophagy [37, 38]. Therefore, detecting autophagy‐associated regulators may be a potential strategy for cancer therapy. In the present study, vortioxetine induced autophagy in AGS cells by increasing LC3 and Beclin‐1, as well as decreasing of P62. Microtubule‐associated protein LC3 and P62 are regarded as important markers of autophagy, usually being employed to evaluate the autophagy level [39]. Beclin‐1 is involved in the nucleation of the autophagic vesicles and could be regulated by autophagy during tumor progression as the tumor suppressor gene [40, 41].

Accumulating evidence has demonstrated that the PI3K/AKT pathway serves as the major negative regulator of autophagy [42], and several drugs can induce autophagy at the early stage of disease, contributing toward resistance to apoptotic death [43]. AKT and downstream mTOR are critical factors in the antagonism of autophagy [44]. Thus, inhibition of PI3K/Akt signaling may represent a potential approach for antitumor therapy. The data obtained in the present study suggested that vortioxetine inactivated the PI3K/AKT pathway by decreasing the phosphorylation levels of AKT and mTOR, as well as cyclin D1. Indeed, correlations have been demonstrated between apoptosis and autophagy; for example, the promotion of autophagy could enhance the apoptosis of GC cells [45, 46]. Hence, we speculated that vortioxetine induced apoptosis and autophagy via activation of the PI3K/AKT pathway in GC cells.

In conclusion, our data show that vortioxetine played a significant role in the proliferation, migration and invasion of GC, as well with respect to inducing apoptosis and autophagy via the PI3K/AKT pathway. The results of the present study demonstrate that vortioxetine might become a new therapeutic strategy for GC. However, the work described comprises a preliminary study conducted at the cellular level, and therefore further investigations regarding the functional mechanism of vortioxetine in different cell lines and animals are still warranted.

## Conflict of interests

The authors declare that they have no conflicts of interest.

## Author contributions

LFZ and GBL contributed to the conceptualization and design of the study, as well as to the writing of the article. GBL, TTW, HLZ, HKW and WS were responsible for performing the experiments and analyzing the related data. All authors read and approved the final manuscript submitted for publication.
